# P-792. Evaluation of the efficacy and safety of a short-course, daily, 4-month regimen including isoniazid, pyrazinamide, rifapentine and moxifloxacin (2HZPM/2HPM) for the treatment of drug-susceptible pulmonary tuberculosis in Taiwan (ESCAPE-TB): A multicenter study

**DOI:** 10.1093/ofid/ofae631.984

**Published:** 2025-01-29

**Authors:** Jia-Yih Feng, Chin-Chung Shu, Shu-Min Lin, Ya-Wei Weng, Jui-Kuang Chen, Kuan-Sheng Wu, Hung-Chin Tsai, Yao-Shen Chen, Hsi-Hsun Lin, Susan Shin-Jung Lee

**Affiliations:** Taipei Veterans General Hospital, Taipei, Taipei, Taiwan; National Taiwan University Hospital, Taipei, Taipei, Taiwan; Chang Gung Memorial Hospital, Linkou, Taipei, Taiwan; Kaohsiung Veterans General Hospital, Kaohsiung, Kaohsiung, Taiwan; Kaohsiung Veterans General Hospital, Kaohsiung, Kaohsiung, Taiwan; Kaohsiung Veterans General Hospital, Kaohsiung, Kaohsiung, Taiwan; Kaohsiung Veterans General Hospital, Kaohsiung, Kaohsiung, Taiwan; Kaohsiung Veterans General Hospital, Kaohsiung, Kaohsiung, Taiwan; Kaohsiung Veterans General Hospital, Kaohsiung, Kaohsiung, Taiwan; Kaohsiung Veterans General Hospital, Kaohsiung, Kaohsiung, Taiwan

## Abstract

**Background:**

The world is not on track to end the TB epidemic by 2030. Efficacious, safe, and shorter treatment regimens may significantly improve treatment success rates, and reduce TB incidence and mortality. The aim of this study is to evaluate the efficacy and safety of a short-course, 4-month regimen (2HZPM/2HPM) in the Asian population.

**Figure:**
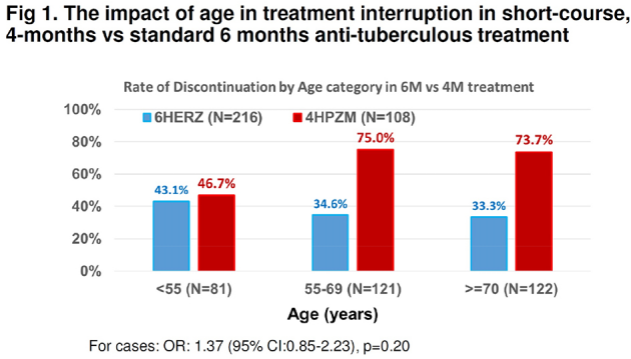

**Methods:**

A prospective, 3-year, interventional study (Jan 2021-Dec 2023) enrolled adults with pulmonary TB, compared to historical controls (1:2 ratio) matched by age and sex, receiving standard 6-months treatment. The primary outcome was TB disease-free survival at 12 months after treatment assignment and proportion of grade 3 or 4 adverse events. A short-course regimen included a 2-months daily isoniazid(H), pyrazinamide(Z), rifapentine(P), and moxifloxacin(M), and 2 months of HPM. Sputum cultures were collected to assess early bactericidal effect at 2 months and to detect relapse at 12 months post-treatment.

**Fig 2.**
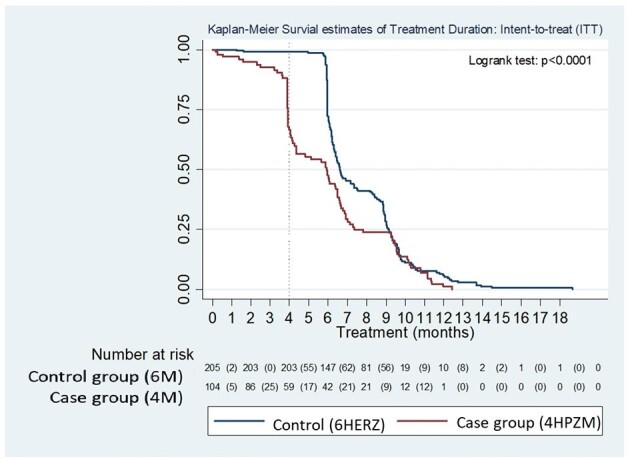
Treatment Duration of short-course, 4-months vs standard 6 months anti-tuberculous treatment: intent-to-treat analysis

**Results:**

109 subjects received short-course therapy, (80 males), with a mean age of 63.4 +/- 15.9 years; and 218 controls. Rates of adverse events were higher in the treatment group but were mostly Grade 1 (54.3% vs 28.7%, p < 0.001), and not significant for Grade 2 (p=0.28), Grade 3 (p=0.59) or Grade 4 (p=0.39). A higher discontinuation rate was found in the treatment group (65.7% vs 35.7%, p < 0.001). Reasons for discontinuation included: hyperbilirubinemia, GI upset, dizziness and others. Currently, 74.1% of the treatment group has completed treatment, 14.8% are under treatment, and 3.7% died, not related to TB. 2-months sputum culture conversion was 86.5 vs 93.4% (p=0.15), in the treatment versus control group, respectively. Survival at 12 months post-treatment was not significantly different between the treatment versus control group (91.7% vs 90.6%, p=0.17). Significant risk factor for treatment discontinuation included age >=55 years (adjusted HR 3.23, 95% CI: 1.27-8.19, p=0.01).

**Conclusion:**

A short-course, 4 months regimen containing HPZM may be as efficacious as current standard, 6-month, anti-TB treatment regimens in Asian patients. Our study demonstrated a high rate of treatment discontinuation in Asian patients. This may be due to the older age of subjects with lower tolerability to adverse events.

**Disclosures:**

**All Authors**: No reported disclosures

